# Piloting an acceptable and feasible menstrual hygiene products disposal system in urban and rural schools in Bangladesh

**DOI:** 10.1186/s12889-020-09413-x

**Published:** 2020-09-07

**Authors:** Farjana Jahan, Md. Nuruzzaman, Farhana Sultana, Mehjabin Tishan Mahfuz, Mahbubur Rahman, Farhana Akhand, Stephen P. Luby, Leanne Unicomb, Peter J. Winch

**Affiliations:** 1 Infectious Diseases Division, Environmental Interventions Unit, International Centre for Diarrheal Disease Research, Bangladesh (icddr,b), 68 Shaheed Tajuddin Ahmed Ave, Dhaka, 1212 Bangladesh; 2 Department of Medicine, Division of Infectious Diseases & Geographic Medicine, Stanford University, 300 Pasteur Dr., L-134, Stanford, CA 94305 USA; 3 Department of International Health, Social and Behavioral Interventions Program, Johns Hopkins Bloomberg School of Public Health, Baltimore, Maryland, 615 N. Wolfe St, Baltimore, MD 21205 USA

**Keywords:** Menstruation, Hygiene, Waste disposal facilities, Toilet facilities, Solid waste, Menstrual hygiene products, Schoolgirls, School enrollment, School dropout

## Abstract

**Background:**

Access to washroom facilities and a place to dispose of menstrual waste are prerequisites for optimal menstrual hygiene management in schools. Like other low- and middle-income countries, Bangladeshi schools lack facilities for girls to change and dispose of their menstrual absorbents. We explored existing systems for disposing of menstrual absorbent wastes in urban and rural schools of Bangladesh and assessed the feasibility and acceptability of alternative disposal options.

**Methods:**

We explored how girls dispose of their menstrual products, identified girls’ preferences and choices for a disposal system and piloted four disposal options in four different schools. We then implemented one preferred option in four additional schools. We explored girls’, teachers’, and janitors’ perspectives and evaluated the acceptability, feasibility, and potential for sustainability of the piloted disposal system.

**Results:**

Barriers to optimal menstrual hygiene management included lack of functional toilets and private locations for changing menstrual products, and limited options for disposal. Girls, teachers, and janitors preferred and ranked the chute disposal system as their first choice, because it has large capacity (765 L), is relatively durable, requires less maintenance, and will take longer time to fill. During implementation of the chute disposal system in four schools, girls, teachers, and janitors reported positive changes in toilet cleanliness and menstrual products disposal resulting from the intervention.

**Conclusions:**

The chute disposal system for menstrual products is a durable option that does not require frequent emptying or regular maintenance, and is accepted by schoolgirls and janitors alike, and can improve conditions for menstrual hygiene management in schools. However, regular supervision, motivation of girls to correctly dispose of their products, and a long-term maintenance and management plan for the system are necessary.

## Background

Lack of menstrual hygiene management (MHM) facilities is a serious barrier to the academic success of schoolgirls in Bangladesh and other low- and middle-income countries (LMICs) [1]. Inadequate MHM facilities in schools pose a major barrier to schoolgirls, making it difficult to maintain proper hygiene and privacy [1]. Global development partners have worked to close the gender gap in education, but insufficient attention has been provided to the specific needs of adolescent girls as they transition to young womanhood within educational institutions [2]. Absenteeism and dropout increase in LMICs as schoolgirls reach puberty [3–5]. Lack of cleanliness in toilets, waste disposal facilities, and separate toilet facilities for female students are important contributing factors to school absenteeism and dropout [2, 4–8].

Lack of facilities for disposal, including water, sanitation and hygiene (WASH) infrastructure, make it difficult for schoolgirls to manage menstruation in schools [9, 10]. The 2014 Bangladesh National Hygiene Survey [11] showed that 40% of schoolgirls missed school on an average of 2–3 days per month due to menstruation. The survey also found that 86% of girls did not change their menstrual absorbent products in school due to lack of privacy or disposal facilities [11]. In many cases, menstrual products are discarded into the toilet, latrines, or ponds, as well as in open areas surrounding the school [12, 13]. Despite the substantial need for a disposal system for menstrual products in schools, there is no such standardized system.

A recent review indicates that provision for disposal of menstrual waste is often neglected, leading to improper disposal and negative impacts on users, the sanitation systems and the environment [14]. There is little discussion or definition of systems for safe disposal and management of menstrual waste in the literature. There is neither agreement on how menstrual waste is classified, nor any clear guidance on how to discard of used menstrual products, leading to inappropriate and unsafe disposal practices [15]. In most instances, the recommendation is that menstrual products be disposed with other solid waste, rather than through toilets and latrines [11–13, 15, 16].

Improper disposal of menstrual wastes also has environmental impacts on soil and water because polyethene plastics in sanitary pads do not biodegrade, and remain in the environment unchanged for many years [17–19]. Lack of proper facilities to dispose of menstrual products leads to improper disposal, and in turn clogging of pipes and system failure [20, 21].

Privacy is an important factor that impacts disposal of menstrual products. Women invest considerable effort in concealing menstrual products, as well as their status as menstruating women [22, 23]. A study in Bangladesh showed that school absenteeism during menstruation was more likely among girls whose schools did not have unlocked or open gender-separated toilet for girls [24]. To support menstruating girls in school, it is important to design toilet facilities to accommodate menstrual hygiene practices and safe handling disposal of used menstrual products. It is also important to know girls’ perspectives and disposal preferences [25, 26].

We could not identify any studies that have piloted and evaluated the acceptability and feasibility of disposal systems in a school context. As a part of a study to develop a school-based MHM intervention package, we assessed alternate methods of disposal for menstrual hygiene products at schools. The findings presented in this paper comprise a sub-study of a project to develop a novel intervention to create a supportive environment in schools for menstrual hygiene management for the Bangladeshi context. In this paper we present findings on 1) characterization of existing disposal systems and identification of four options for improved disposal systems, 2) piloting four alternative disposal systems in four schools to assess acceptability and feasibility, followed by 3) implementation of one of these options in an additional four schools.

## Methods

### Study approach

This study was conducted in three phases:1) pre-intervention, 2) intervention design, and 3) intervention implementation. The pre-intervention and intervention design phases were conducted in four ‘formative schools’, and intervention implementation occurred in a further four schools, or ‘pilot schools’. To better understand disposal practices and preferences in a variety of settings, we selected co-educational schools with grade 1–10 from urban and rural areas of Bangladesh. We selected four schools from Dhaka metropolitan city to represent an urban setting and four schools from Manikganj district to represent a rural setting. The study participants were students of grades V to X, teachers, and janitors.

### Pre-intervention: identifying MHM facilities and current disposal practices among girls in school (4 months)

The field team conducted in-depth-interviews (IDIs) (48) with randomly selected girls, participatory ranking exercises (4), drawing exercise (4), a vignette exercise (4), and key informant interviews (KIIs) with janitors (4) to identify girls’ common practices for disposing menstrual products, sources of stigma, perceptions and attitudes regarding puberty and menstrual hygiene management and their preferences for disposal systems. The team also collected information on current menstrual product disposal management in schools and the role of janitors in waste management. Finally, the field team conducted spot checks of facilities (4) related to water, sanitation, and menstrual absorbents disposal facilities to assess the existing MHM situation.

Four vignette exercises were conducted with girls to explore puberty and menstruation related perceptions, knowledge, barriers, stigma, shame, guilt, and embarrassment. This was also to ensure menstruating girls’ voices and participation in designing the intervention, and to understand the cultural and school context that contributes to girls being stigmatized about menstruation. In each vignette exercise 25 girls participated.

During participatory ranking exercises, fieldworkers displayed available menstrual absorbents and pictures of disposal bins in order to understand girls’ preferred options for menstrual hygiene management products and disposal system.

For drawing exercises, fieldworkers provided girl students a drawing paper and color pencils, and instructed them to put together their preferred choices for menstrual hygiene management that they perceive is important to keep them in schools during menstruation. A total of 48 girls participated in the ranking and drawing exercises. For selecting the participants of vignette, drawing and participatory ranking exercises field researchers collected the roll number of present students from class V to X on that day and then from each class equal number of students were selected. The students who wanted to participated willingly were selected.

### Intervention design: identifying and initial piloting of four disposal systems (3 months)

In this phase we identified four disposal options and assessed the acceptability, preferences, and feasibility of the initially piloted disposal systems in four different schools to facilitate final selection of one intervention design.

**Fig. 1:**
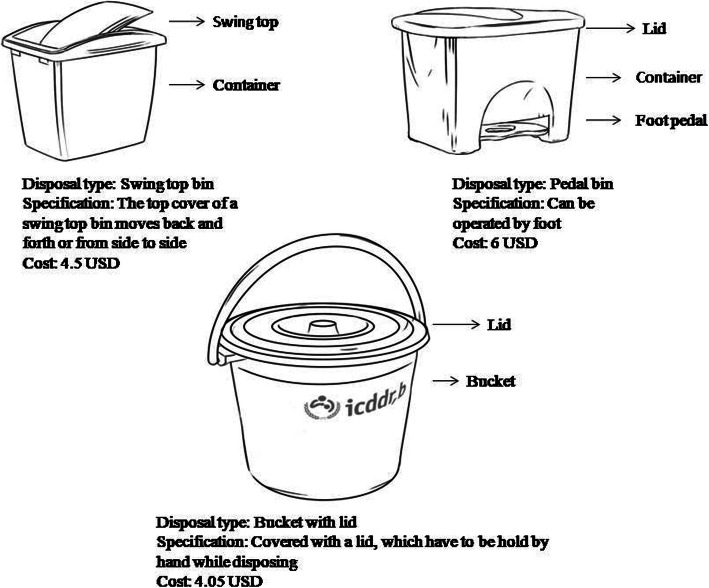
Disposal system options used in the pilot schools of Dhaka and Manikganj in Bangladesh, April–June, 2017 (Source: icddr,b; illustrated by project staff)

**Fig. 2:**
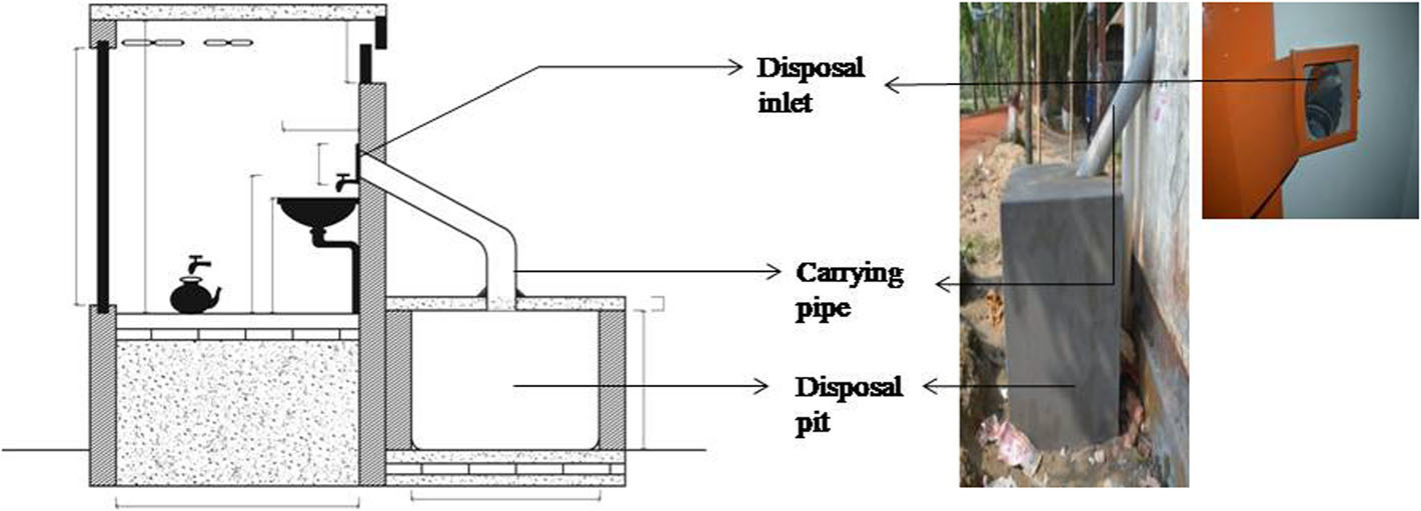
Piloted and implemented chute disposal system in pilot and implementation schools of Dhaka and Manikganj (Source: DSK and icddr,b, permission obtained from DSK to use the chute disposal design)

We conducted a day long intervention development workshop at icddr,b offices in Dhaka to identify four potential menstrual products disposal systems. The workshop convened stakeholders from the Ministries of Health and Education, the Public Health Engineering Department, non-government organizations working in improving MHM and WASH facilities in schools, and other institutions. During the workshop, we shared findings from the pre-intervention phase and sought recommendations for improving MHM in schools from the stakeholders. Based on the current situation and available feasible options for disposing menstrual products, the workshop participants suggested disposal options for initial piloting. The four recommended disposal systems (Figs. 1 and 2) were initially each piloted in a different school in Dhaka and Manikganj district for 3 months. The final ranking by girls, teachers, and janitors after three-month period of piloting used pictures and verbal descriptions of the disposal systems by the field researchers.

Field researchers conducted IDIs with 139 girls from four schools, natural group discussions (NGDs) with teachers and janitors (4), and spot checks (12) of the toilets and washrooms for the four disposal options. We collected information on types of waste disposed, functionality of the disposal system, perceived benefits and barriers of the installed disposal system, and damage (e.g. broken or missing components). The IDIs and NGDs lasted for 60–80 min. Field researchers described all four disposal systems with accompanying illustrations to girls, teachers, and janitors during a participatory ranking exercise after 3 months of initial piloting, and asked all the participants to rank the disposal systems according to their first, second and third most preferred options. A total of 163 (139 girls, 20 teachers and 4 janitors) participants from four schools participated in the ranking exercise. Girls, teachers, and janitors ranked the four disposal systems according to their preference. We then combined the ranking results from four schools and identified the most preferred option.

### Intervention implementation: final implementation of preferred disposal system (6 months)

**Fig. 3:**
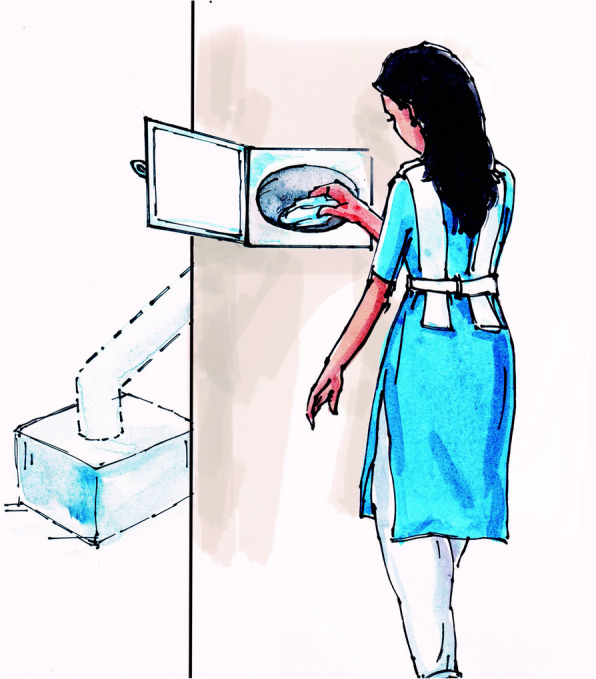
Visual aids to display menstrual hygiene management and disposal technique in the implementation schools of Dhaka and Manikganj, 2017 (Source: icddr,b; conceptualized by project staff and illustrated by Artist Sunil Kumar)

Based on the initial pilot study findings, we implemented the chute disposal system (Fig. 2) in four implementation schools. We provided visual aids (Fig. 3) to demonstrate MHM and menstrual products disposal procedures, and instructed students to wrap disposable pads with paper (newspaper or old writing paper) before disposal. Field researchers conducted group discussions to introduce the intervention components to students, teachers and parents and formed a gender committee in each pilot school to facilitate supervision of overall intervention activities and maintenance of toilet facilities along with the chute disposal system, including supply of paper for wrapping the used absorbents. Each school formed their own gender committee. The gender committee of each school included representatives from each grade (V to X) of the schools, teachers, members from the school management committee, members from the parent-teachers’ association, and janitors.

The gender committee held a meeting once a week with its members to evaluate how the intervention is being implemented and if they faced any challenges.

**Fig. 4:**
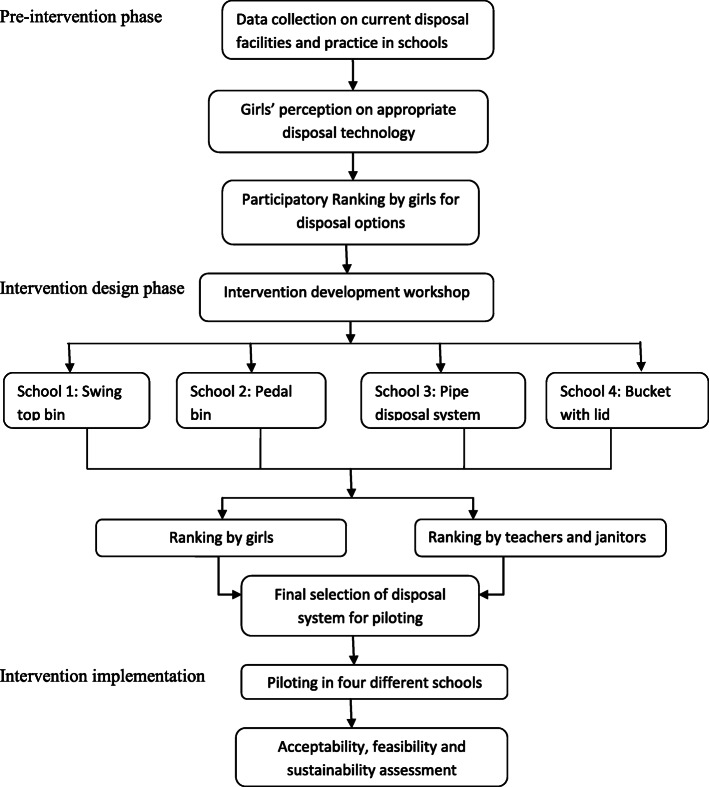
Flow chart for selection of disposal technology options for testing and piloting

The field team conducted monthly fidelity assessments which include spot checks and interviews to determine whether the intervention had been delivered as planned and conducted IDIs to assess the acceptability, feasibility, and potential for sustainability of the chute disposal system. The interviews were conducted monthly, starting from 1 month after installation at the schools, during lunch break or before the start of class. Field researchers conducted 100 IDIs (5 IDIs per each school per month for 5 months) with randomly selected girls, 20 IDIs with janitors, and 20 spot checks of facilities during the intervention period to assess conditions of the toilet and disposal system, usage by girls, perceived benefits, perceived barriers, maintenance, and suggestions for further improvement of the chute disposal system. The process of development and selection of a final candidate disposal system is illustrated in a flow chart (Fig. 4).

### Data analysis

We conducted thematic analysis of the qualitative data following a process that was both deductive and inductive. Inductive codes predominated in the earlier phase of research, and were generated through close reading of selected transcripts, and then the codes created were applied across all transcripts. Deductive codes predominated in the later phase of research, where the disposal options had been defined, we sought to examine the usage, acceptability, and feasibility of the four initially piloted disposal systems. Researchers recorded IDIs, KIIs, NGDs, and vignette exercises using digital audio recorders. Later, we transcribed the audio recordings verbatim into Bengali in the word processor and coded using the qualitative data analysis software Atlas.ti (version 5.2). For coding in Atlas.ti, we prepared different code lists for all of the different categories of informants based on the major themes and objectives of the research and brainstorming the information researchers found during data collection.

Then, we summarized the coded data into English. We analyzed the drawing exercises manually based on the images that participants created to express their ideas about what a supportive environment for attending school during menstruation would look like. Field team made notes during the ranking exercises, and we summarized the notes into a ranking table in MS-Word. The field team collected the spot check observations with a structured form and analyzed those data in the quantitative data analysis software SPSS. Finally, we summarized the findings from the coding by major themes.

During interpretation of the data, we have compared different IDIs conducted with girls, teachers, and janitors, and also the NGD findings and participatory exercises with each other findings for consistency. Multiple researchers were involved in coding data to ensure the validity.

The data collection instruments used for this study are available as supplementary file (Additional file 1).

### Ethical approval and consent to participate

The protocol was reviewed and approved by icddr,b’s institutional review board (IRB). We obtained permission from the Divisional Secondary and Primary Education Office at Dhaka, to conduct intervention activities in the school. All participants provided informed written consent or assent (< 16 years old) for participation in the study. We took parents’ consent for participants who were < 16 years old. Field researchers explained study objectives, activities, anonymity, and confidentiality to the participants and assured them that they could withdraw anytime. Our study did not carry any physical or psychological risks to the participants.

## Results

A total of 468 individuals, including students (419), teachers (21), and janitors (28) took part in the study. The mean student age was 14.2 years (SD1.39; Table 1). We present the results from each phase by theme.

**Table 1:** Socio-demographic characteristics of the study participants of formative, pilot and implementation schools of Dhaka and Manikganj, 2016–2017

Characteristics	Pre-intervention n(%)	Intervention design (Initial piloting) n (%)	Final intervention implementation n(%)
**Type**	*N* = 185	*N* = 151	*N* = 120
Student	168 (90)	139 (92)	100 (83)
Teacher and janitors	17 (10)	12 (8)	20 (17)
**Sex**			
Female	127 (72)	149 (98)	120 (100)
**Education**	*N* = 168	*N* = 139	N = 139
Primary	5 (3)	4 (3)	–
Secondary	163 (97)	135 (97)	100 (72)
**Parent’s education**	*N* = 336	*N* = 278	N = 120
Primary or no education	99 (55)	103 (48)	47 (30)
Secondary	120 (45)	93 (43)	89 (58)
Higher secondary and above	52 (8)	20 (7)	17 (12)
**Parent’s occupation**	*N* = 336	*N* = 278	*N* = 200
Housewife	133 (77)	96 (43)	95 (45)
Small business	51 (4)	27 (12)	42 (20)
Non-government job	59 (7)	33 (15)	33 (16)
Work abroad	19 (2)	16 (7)	9 (4)
Other^a^	34 (6)	52 (23)	31 (15)

^a^Shopkeeper, farmer, tailor, rickshaw puller, barber, day laborer, professor, unemployed

### Findings from pre-intervention phase: current practices and barriers to dispose used menstrual products at school

During pre-intervention phase IDIs, out of 48 girls, 30 reported that they miss school during menstruation due to lack of supportive facilities in school. About two-thirds of the interviewed (32) girls mentioned that they did not change sanitary pads during the 8 h of their school day. Out of 48 girls interviewed, 29 girls mentioned that they threw used pads out of toilet windows toward the back of the school compound. One girl (grade VI, urban school) said*: “As there is no disposal bin in the school, I can’t change the used pad. It causes me discomfort, and I always feel I might get a stain or that I am already stained! Still, I can’t change my pad, I would rather wait until school hour is over. However, if I feel too uncomfortable, I ask for leave from the teachers, and return home to change pads.”*

Girls from both urban and rural schools mentioned that they prefer to not use school toilets, especially during menstruation, as they 1) require standing in a queue, 2) there are no facilities to change or dispose of the used products, 3) fear of being teased by others, 4) tension about whether they can make it back to class in time due to the long queue, 5) toilet does not lock properly, 6) lack of sufficient ventilation and light, 7) lack of soap and water supply, and 8) offensive statements on walls of toilets.

During facility spot checks we found only three waste bins in the 28 functional toilet cubicles checked in four schools, though the bins were not designated for sanitary product disposal. The sewage line was blocked in two schools. According to teachers and janitors, the sewage line was blocked because girls were disposing of sanitary products or rags directly into toilet bowls. A hired sweeper had to be brought in to remove the blockage, which was a financial burden for the schools. One female teacher (50–60 years, urban school) said: *“Sometimes girls dispose of sanitary products inside the latrine due to the lack of disposal bins. Thus, we suggested them to carry a plastic bag to store used sanitary products and dispose of them later while they reach home”.*

During one KII, a 50–55-year-old janitor from one urban school mentioned, “a sweeper at an urban school, after clearing the blockage of sewage line, reported that the sewage line was blocked because girls were disposing their sanitary products or rags into the toilet”.

The girls from rural schools commonly mentioned that they do not use menstrual cloths during school hours as there is no room for changing and washing. One girl (grade X, rural school) said: *“Generally I use cloth, but I can’t change and wash the used cloth in the school. So, I prefer wearing pads during school hours, though it is costly!”*

The girls we interviewed who use menstrual cloths did not reuse them, but rather buried or disposed of used cloths and pads because they believe heavy bleeding or abdominal pain might occur if they wash and dry the cloths in the open. One girl (grade V, urban school) said: *“We do not dry rags outside to avoid exposure to bad airs (malevolent spirits) that might bring us serious illness and hospitalization. Our elders prevent us from talking to boys during menstruation as they are afraid that we might get pregnant. They also prevent us from going out at noon and in the dark as the malevolent spirit can cause us harm.”*

During the spot checks of the facilities, fieldworkers found out of 18 functional basins 4 had soap, and those toilets were designated for teachers use only. The majority of the students reported that the school had intermittent water supply.

### Vignette, drawing and participatory exercises: girls’ perception regarding menstruation and preference for menstrual hygiene management facilities

During vignette exercises out of 100 participating girls, most (86) mentioned that their family members, relatives, and neighbors were reluctant to discuss puberty and menstruation related issues as they considered 1) it is the social norm to keep it as private, 2) girls are too young to be discussing such topics, 3) such discussion would increase their curiosity and interest in the opposite sex, and 4) menstruation in particular is a matter considered deeply shameful. Most girls (76) also reported that their school curriculum lacks sufficient information about puberty and menstruation, so that they fail to develop a clear understanding about MHM. Additionally, most of the responsible teachers avoid taking responsibility for puberty and menstruation-related classes and advise students to read about these topics at home. Almost all the participating students perceived puberty and menstruation to be a matter of shame, and that, especially menstruation-related matters shall not be discussed with opposite sex to avoid teasing. Girls reported that the schoolboys teased them for staining their clothing, and laughed at them at the point in the class when teachers were taking puberty and menstruation, or when they went to purchase sanitary pads from the janitors.

One girl at grade *VIII* in a rural school said: *“We do not pay attention to the physical education classes where ma’am talks about puberty or menstruation because we feel uneasy, and it is embarrassing talking about the topic. I don’t understand why ma’am does not feel uneasy! Boys laugh at us at class while she discusses such topics by asking how did we feel about the topic!*”

**Fig. 5:**
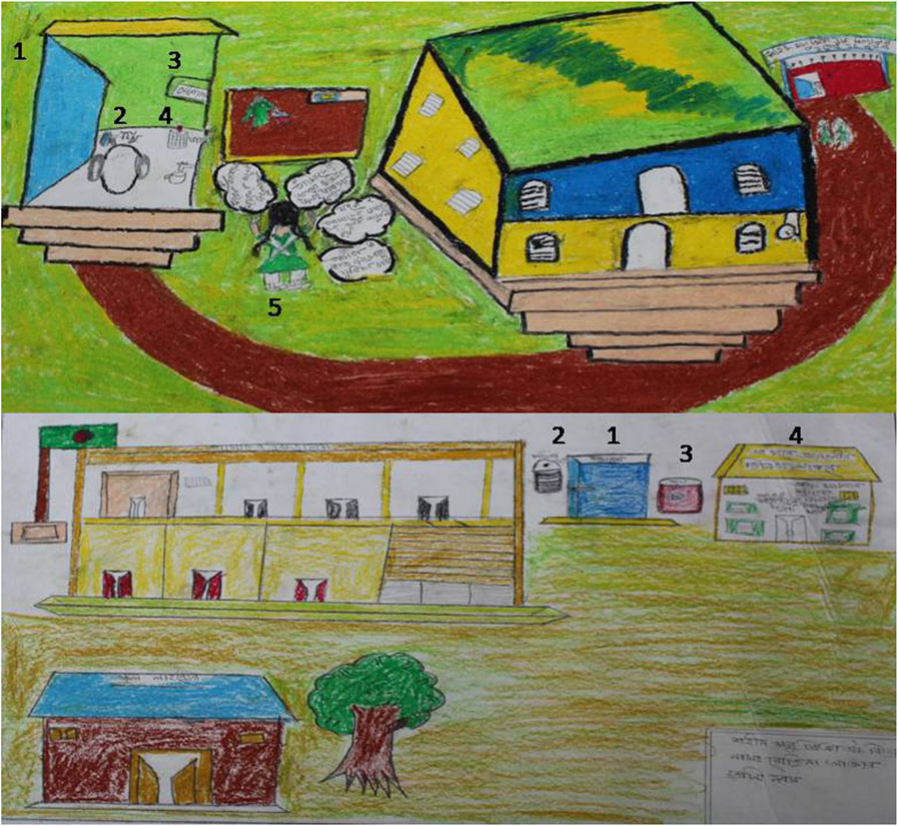
Drawing exercises by schoolgirls at pre-intervention phase in four schools of Dhaka and Manikganj district, 2017(Source: icddr,b)

During drawing exercises, girls drew the ideal facilities that should be present in school that will make them more comfortable to attend schools during menstruation (Fig. 5). The most commonly recommended facilities in their drawing were: separate toilet and washroom for girls, provision of toilet paper, sanitary pad and disposal bin in the toilet, stored water and soap for handwashing. Along with menstrual products and private spaces, a disposal system specifically designated to dispose of menstrual products was frequently recommended by girls during participatory exercises (Table 2).

**Table 2:** Recommendations by girls through drawing and participatory ranking exercises to create supportive menstrual hygiene management environment in formative schools of Dhaka and Manikganj, 2016

Facilities	Recommendations	Quotations
**Menstrual hygiene management Products**	During the participatory exercises, all the school girls voted their preferred choice of sanitary products and ranked sanitary pads with belt as first choice, sanitary pads with underwear as 2nd choice, and rags as third choice. The majority of the girls preferred free, or at least subsidized (per piece US$ 0.06 to 0.13) provision of sanitary pad stocks with female teachers in schools.	One girl aged 12 years at grade *V* in a rural school said: *“It would be good if the school can have pad arrangements as we need the product. Any of the female teachers can keep those with them, and we can collect from her.*”
**Disposal bin**	During the participatory exercises, all the school girls voted their preferred choice of disposal system and ranked paddle bin with lid as first choice, and swing top bin as second choice. Girls asked for disposal bin with lid to dispose used pads. They suggested all the girls shall wrap the pads, may be with newspaper which is low-cost, before disposal. The bin can also be wrapped with a plastic to avoid staining the bin, and the disposed pads finally can be buried or burned. Regarding maintenance of those bins, they named janitors, and also suggested that a girl from each class can lead the responsibility.	One girl aged 15 years at grade IX in a rural school said: “*It is necessary to have disposal bins that need to be emptied and cleaned. School authority, it would be good if the school authority manages to clean the bins. Otherwise, we have to take the responsibility. For every class there is a captain and I will be agreed upon taking the responsibility to clean the bins.”*
**Toilet facilities**	Girls preferred gender segregated toilet facilities, and suggested to increase the number of toilets with the provision of soap, regular water supply and cleanliness.	One girl aged 16 years at grade X in a rural school said: *“I ask for 5 min of leave from class to go to the toilet, though it takes more time as we have to wait in a long queue. We only have two toilets for girls, and it would be great if we could have more toilets!”*
**Privacy**	Girls also asked for a separate room for girls that will provide them privacy, and an access to take rest, manage sudden menstruation or sickness.	One girl aged 15 years at grade *VIII* in an urban school said: *“We will be inspired to attend school during menstruation if our teachers can ensure a supportive environment in the school. There shall be a separate room for us to give us a space.”*
**Healthcare options**	Girls mentioned that sometimes they feel abdominal pain for which they suggested for the provision of pain relief medications, and a doctor or counselor to prepare girls in menstrual hygiene management.	One girl aged 15 years at grade VII in an urban school said: “*You can arrange primary medical care facilities at school from where we can collect medicines if needed. We also need someone like a counselor to discuss our frustrations and frightens”.*

### Usage, acceptability, and feasibility of the four initially piloted disposal systems

From pre-intervention phase study findings, we identified three disposal systems readily available in local markets that required daily emptying: a swing top bin, a pedal bin, and a bucket with lid (Fig. 1). During the intervention development workshop, participants identified another option: a chute disposal system, where sanitary pads can be directly disposed into a deep covered pit outside the toilet, accessed through a covered inlet inside the toilet (See Fig. 2). The chute disposal system was specifically designed for disposal of menstrual products by Water Aid and Dushtha Shasthya Kendra (DSK), two non-profit organizations.

While the first three disposal options are temporary, of small capacity (10-30 L), portable, and less expensive (4–6 USD), the chute disposal system is more permanent, of large capacity (765 L), and requires some capital investment (87 USD) and long-term maintenance plan (emptying and decomposition) by the school.

IDIs with girls and NGDs with teachers and janitors from the four pilot schools revealed that almost all were aware of the presence and location of the newly allocated disposal systems. Out of 139 (37 for pedal bin, 36 for swing top bin, 32 for bucket with lid and 37 for chute disposal) interviewed girls, 46 girls reported using the disposal system in school. Out of these 46 girls, similar numbers (13, 10 and 11) of girls used swing top bins, pedal bins and chute disposal, while the bucket with lid was used by only one girl. Among the 50 girls who reported not using any disposal system, most stated that they used reusable cloths, and that they carried these cloths back home for washing and drying. Some girls (8) reported that they had not become habituated to the school toilets as they felt disgusted by them. They found the school toilets to always be dirty, poorly-lit, and have a bad odor. Thirty of the girls reported feeling disgust if they touched the lid of the waste bucket by hand, and that there were no tissue papers or soap to wash hand after touching the lid. They also mentioned, sometimes the janitors did not empty the bins, making the toilet unclean.

Most girls (129) liked all three disposal systems (pedal bin, swing top bin and chute) in schools, but were unaware about the maintenance issues resulting from improper disposal of absorbent materials. Girls recommended provision of visual aids to remind them and to display the maintenance procedure.

All chute disposal system users knew about the disposal procedure: wrapping the used pad with paper and then disposing it directly in the pipe. Out of 11 users of chute, 5 recommended modification of chute. They suggested the inlet of the piped disposal system should be in lower position. Two users of pedal bin and swing top bins suggested the size of the other bins should be bigger. They also recommended placement of the chute in another toilet which reserved for use by girls. One girl from grade *VII* in rural school reported*; “This cubicle is not in a good condition, sometimes it is blocked, but the other cubicle is in good condition. You can install a bin there”.*

A 40–50-year-old janitor of a rural school stated; *“There are two toilets but the piped disposal system is in one toilet, some girls also disposed [materials] in other toilets, if pipe could be connected in both toilets it would be better”.*

The janitors in the schools where we initially piloted the three temporary disposal options reported that it was a burden for them to empty the bins every day. There was only one janitor per school and they mentioned that apart from cleaning the bins in the toilets, she also had to clean the classrooms, restock supplies in the classrooms, and respond to other instructions from individual teachers. She also mentioned that girls do not dispose of the used absorbent products properly in the bin, sometimes they would fall on the floor.

The janitor of school with chute disposal mentioned that its installation had been very helpful, for she did not need to clean it regularly. But sometimes she had to clean the inlet, as the girls did not properly dispose of the product into the pipe.

The janitors were asked where they ultimately dispose the waste collected in bins inside the toilet cubicles. They reported that they threw it on the trash heap in front of the schools, which subsequently was collected when municipal waste pickers passed by. In case of chute disposal system, the school management committee planned for chemical decomposition of waste in the pit. Once the pits get filled up, they will put chemical inside the pit for decomposing the waste and will clean the pit of decomposed waste.

### Perceived benefits and barriers of the piloted four disposal system options by girls

During initial piloting, girls reported many benefits of the disposal systems. Most users of the pedal bins, swing top bins, and chute disposal systems reported the benefit of not needing to touch the disposal system with their hands. They reported improved school cleanliness after installation of the disposal system. The main barrier they faced was that school toilets were not regularly cleaned. This created a bad odor, hence they avoided school toilets as much they could (Table 3).

**Table 3:** Benefits and barriers of four different piloted disposal systems by girls of pilot schools in Dhaka and Manikganj, April–June, 2017

Perceived benefits and barriers	Frequency of Response *N* = 139	Relevant quotations
**Benefits**		
• Toilet facilities are cleaner after installing disposal systems.	90	“Yes, It’s good for everyone. Toilets are cleaner than before. We don’t have to throw it anywhere else”. (12 years, grade VIII, rural school)
• Girls do not leave school to change their menstrual products.	60	“*It is far better. Because, earlier, there were basket[s] and when the boys noticed us throwing used pads, we would feel ashamed. Now the bins are covered. It is good, it is easy to dump pads”.* (15 years, grade X, chute disposal user, urban school)
• Disposal systems easy to use	35	
• Girls are comfortable to manage their menstruation at school	30	*“Earlier we used to leave school and go home to change our pads when it is overflowing, now we change it in school.”* (13 years, grade VIII, rural school)
• Disposal took less time- girls do not need to find any place to secretly dispose their products	65	“*Unlike traditional waste baskets, it is easy to use. There is no difficulty in using it, [it’s] easy to use and the process is good. It is not even disgusting”.* (11 years, grade VI*,* urban school, swing top bin user)
• Disposed materials are not visible from outside, which is comforting to girls as well as janitors.	16	
**Barriers**		
• Toilets are not clean enough to visit	45	“*I do not go to [the] toilet because of bad odor. So, I am not sure what kinds of disposal bins are located there”*. (15 years, grade X, urban school)
• Pedal bin, swing top bin and bucket with lid requires regular cleaning	25	“*The inlet from the chute disposal system was too high. Short girls may face problem disposing in the pipe”. (12 years, grade VII, urban school)* “*There was a pad stuck in the mouth of the bin. The students do not know how to resolve the problem. I do not know either”.* (14 years, grade IX, urban school, chute disposal user)
• Inappropriate location of the disposal system	12	
• Quick filling of swing top bin, pedal bin and bucket with lid	11	
• Improper disposal of sanitary pads into the bin- some girls did not wrap the pads before throwing.	3	*“Girls did not dispose [waste] properly; they did not wrap the pads, so the pads got stacked [up] in [the] opening of the pipe”.* (Janitor, urban school)

Comparison of the four initially piloted disposal systems on the basis of preference, capacity, complexity, operation and maintenance is shown in Table 4. The temporary disposal systems are of low capacity (10-30 L), low cost (4–6 USD), easy to install (no technical assistance required), can be placed anywhere, require little to no hand contact, however their durability is low, and they require regular emptying. On the other hand, chute disposal system is of large capacity (765 L), relatively high cost (87 USD), needs more space and technical assistance is necessary for installation, but it is relatively permanent, does not require regular emptying, and requires more supervision for uninterrupted operation. The measurement of the pit connected to chute is 3 ft*3 ft*3 ft. The length of the pipe depends on the location of the toilet and the pit.

**Table 4:** Comparison of the piloted disposal systems in four pilot schools of Dhaka and Manikganj (April–June, 2017)

Indicator	Swing top bin	Pedal din	Bucket with lid	Chute disposal system
Capacity (In liter)	Small (10 L)	Small (10 L)	Small (30 L)	Large (765 L)
Cost (In USD)	4.5 USD	6 USD	4.05 USD	87 USD
Need of technical assistance while installing	Easy to be placed, no technical assistance required	Easy to be placed, no technical assistance required	Easy to be placed, no technical assistance required	Need some technical assistance to install (to build pit, make to inlet and placement of connecting pipe)
Requirement of space to install	Need less place, can be placed anywhere	Can be placed in a narrow space	Can be placed anywhere	Relatively larger space (9 square feet) required to build up the pit.
Handling	Requires no or minimum touching to dispose	Requires no touching to dispose	Requires minimum touching to dispose	Requires touching to open the lid
Visibility of pre-disposed waste	Waste is visible while disposing	Waste is visible while disposing	Waste is visible while disposing	Waste is not visible while disposing
Durability	Low	Low	Low	High
Maintenance	Requires regular maintenance	Requires regular maintenance	Requires regular maintenance	Does not require regular maintenance
Ultimate disposal	Requires secondary disposal	Regular secondary disposal is needed to maintain cleanliness	Regular secondary disposal is needed to maintain cleanliness	Disposed materials can be chemically decomposed or incinerated within the pit when it gets filled up

### Disposal options ranking and selection of one option for final implementation

All the girls, teachers and janitors ranked the chute disposal system as the most preferred and accepted disposal system. Out of 163 total participants, 101 participants ranked pedal bin as their second option, and 62 participants ranked swing top bin as the second option. No one voted for the bucket with lid. Reasons given for ranking the chute disposal system in the first position were that it can be used for a long time, does not need regular maintenance, and reduces odor. Due to their small holding capacities (10-30 L), the other three disposal options filled up quickly and needed to be emptied and cleaned frequently. The chute disposal had a large capacity (765 L) projected to take 2–3 years to fill (DSK, personal communication), thereby not requiring regular maintenance. It is a durable structure, and difficult to damage or break. The schoolteachers also explained that if the disposal systems became damaged easily, they would not be able to replace it quickly due to budgetary and procurement constraints in government schools. All materials need to be procured by informing school authorities, which is time consuming. Therefore, it is better to have a durable and relatively permanent disposal system in schools, even if it needs some capital investment.

While asked about long-term maintenance, the head teachers (4) of the schools mentioned, school management committee will monitor these activities and they can keep an amount of fund for maintenance of disposal systems. Janitors also appreciated the chute disposal system with a larger capacity, as it would require less frequent emptying. School committees reported difficulty to change or empty disposal systems regularly as there are resource constraints. One male teacher (55–60 years, rural school) stated; *“Other disposal bins could be broken or be damaged, the cover could be damaged also. If the girls disposed menstrual products in broken bins, it can spread foul smell and create nuisance”.* In reference to the chute disposal system, another female teacher (40–50 years, urban school) asserted that, *“It did not require regular cleaning; it will last a long time. Girls did not feel shy as the inlet of the chute is within the toilet and nobody could see her disposing”.*

The majority (132) of the girls mentioned they ranked chute first, as 1) the chute has larger space they will not need to ask the janitors to empty the bin often, 2) other bins are made of plastic so can be broken anytime and it’s not feasible to ask teachers to provide disposal bin by themselves, 3) the wastes are not visible at all while disposing in the chute, for which they don’t feel disgust to use it.

A disposal system that does not require frequent replacement and cleaning like other disposal options available in the market was most preferred by all participants. The chute disposal system was selected for piloting in four other schools.

### Piloting chute disposal system in schools: acceptability and feasibility

About one fifth (21) of the interviewed girls used the chute disposal system once it was installed. The main reason for reporting non-use was that girls (60) preferred re-usable cloth pads to disposable materials, and therefore had no need of a disposal option of absorbent products. Some (7) of the girls mentioned that they felt no need to change their absorbents because of shorter length of the school day during school examinations. Almost all (97) of girls did not report any barriers to using the chute disposal system. Almost all (96) found it helped them to feel comfortable to attend the schools during menstruation.

### Perception of janitors in disposal system maintenance

Three of the four interviewed janitors from four schools reported no problem with maintenance of the chute disposal system. One janitor (50-60 years, rural school) reported one event when she faced a problem with maintenance: *“Some girls did not throw the pads properly through the pipe; rather they placed the pads in the opening without wrapping them first, [so] I had to push the pads through with a stick to clean the inlet.”*

All the formative and pilot schools had only one janitor who is responsible for multiple tasks; maintenance of toilets is only one of them. Janitors are responsible for school cleaning, arrangement of snacks for students, and carrying school files and register books to teachers. Janitors reported that it is a challenge for them to empty and clean menstrual product disposal containers on a regular basis, resulting in unclean school toilets.

## Discussion

In this study we identified school menstrual absorbent disposal practices. We identified four disposal systems and pre-tested them to select one acceptable and feasible disposal system for further piloting. We found that the chute disposal system was acknowledged as potentially sustainable and easy to use and maintain, addressing the lack of disposal facilities for girls in schools in both rural and urban Bangladesh. Our study findings broadly had three implications discussed below.

### Disposal facilities are an essential part of school sanitation to ensure menstruating girls’ comfort in schools

Our findings align with other research in this setting that girls miss school during menstruation due to lack of supportive facilities. Girls do not change and dispose of their used menstrual products at school due to lack of disposal facilities which is consistent with survey and study findings conducted in Bangladesh, Malawi, Ethiopia, and Uganda [11, 27–29]. We found that girls dispose of the used sanitary pads improperly by throwing in the garbage, soil and sometimes in the pond. This improper disposal will have a negative impact in our environment [18, 19]. Study findings revealed that improper disposal of menstrual waste causes blocked sewer lines, which imposes a financial burden on school authorities and consequently on the government. This has similarity with findings reported in Tanzania, Kenya and Philippines: in Tanzania, the Dar es Salaam Water and Sewerage Corporation records an average of 150 blockages per month due to menstrual waste, costing USD 25,000 per month to rectify [20].

Girls recognized disposal facilities as an essential part of MHM in school. Girls recommended clean, hygienic, separate toilets with appropriate disposal facilities and proper operation and maintenance. Water Aid, UNICEF and WSUP (Water and Sanitation for the Urban Poor) published a female-friendly toilet guideline on the basis of recommendations from women and girls of Bangladesh, India, and Kenya, which recommends that toilets should have separate facilities for changing and disposing of menstrual products [30]. This is also consistent with study findings from Connolly and Sommer, where Cambodian school girls recommended similar facilities for their menstrual hygiene management in schools [31].

During our pilot, girls reported increased comfort in attending school during menstruation when they could change and dispose of their menstrual absorbents in school, in line with study findings reported by Tegegne and Sisay from Ethiopia, where girls dropped out from schools after menarche due to absence of separate sanitation and disposal facilities [27].

Girls felt disgust towards menstrual waste and wanted to dispose of it quickly and secretly. Girls liked disposal systems with covers that reduced odor, kept the toilets clean, and eliminated the need for hand contact. A study conducted in New Zealand also found that girls’ fears of their menstrual waste being seen if not covered led to many girls disposing of waste in the toilet because it removed the waste quickly and was not likely to be seen [32]. A study from India suggested that special covered bins should be installed to manage menstrual waste, as girls found it unpleasant when menstrual waste was disposed in uncovered bins due bad odor and sight [33].

### Operation and maintenance of disposal system is a major concern

According to the WASH in schools guidelines for LMICs, toilets and disposal systems should be regularly maintained [34], a challenge in low resource settings. For any disposal systems maintenance was a paramount concern, and in this study the option requiring less maintenance was chosen over others. Janitors play an important role in the maintenance of sanitation facilities including disposal system [35]. The expressed preference for the chute disposal system due to its low requirement for routine maintenance complements findings from other studies conducted in the USA and UK that identified maintenance as a key barrier [36, 37]. Once installed, a chute disposal system is assumed sufficient without cleaning and maintenance for at least 2 years. However, chute disposal systems need more planned management compared to other disposal options, including capital investment for installation. The chute disposal system requires a relatively large space (9 square feet) to install than other disposal systems and cannot be replaced easily once full. We installed the chute disposal systems behind toilet, as there was sufficient space there. It will be difficult for schools without any available space. However, it reduces the burden of frequent maintenance and our findings indicate that it may be used more regularly than other disposal systems. That said, schools choosing to install a chute disposal system will need to incorporate supervision and training for students to avoid improper disposal of waste and subsequent pipe blockages. Chute disposal systems may also help reduce improper management of menstrual waste once it enters the main waste stream. Our study findings showed, for temporary disposal bins, the wastes were ultimately disposed in the garbage with other solid waste. A report from India recommended menstrual waste should not be disposed of along with domestic waste, as municipal waste pickers find sanitary pads disgusting to touch and may strike to not pick up unwrapped sanitary pads because they believe they can fall sick from handling menstrual waste [38]. This emphasizes the need for school authorities to ensure supply of paper to wrap and dispose of menstrual absorbents, but also for schools to develop a long-term disposal plan for systems such as the chute disposal; it will be essential to plan a final disposal place, and the method and manpower needed to empty it.

### Janitors play an important role in the maintenance of disposal systems

Janitors are key individuals in the maintenance of disposal facilities as well as toilet facilities. Piloting or scaling up any disposal facilities should include recommendations from janitors. A respiratory hygiene promotion study conducted in Bangladesh formed a hygiene committee for the operation and maintenance of intervention component, and janitors were a key contributor to that committee [39]. As the disposal facilities are located in the toilet block, usage of disposal options depends not only on its availability but also on toilet cleanliness, for which the janitor is responsible. Due to over workload janitors are not willing to clean the toilet regularly. Another contributing factor is the low salary paid to janitors. Increasing remuneration or increasing the number of janitors can be one of the possible ways of improving toilet maintenance. Some of the studies showed students on their own initiative can take responsibility for toilet cleanliness [35]. Our study findings suggested that some girls did not use the disposal system because the toilet was dirty and had a bad odor. Each school had at least 3–4 toilet cubicles that janitors had to maintain along with other official tasks. In order to make disposal systems a sustainable option, increasing the number of janitors or increasing remuneration and motivation will also necessary.

### Limitations

During initial piloting we did not install all the four disposal systems in each school, due to resource constraints and space problem in school premise. Rather, we piloted one disposal system in one school, and, in order to explore other schools’ thoughts on the systems they had not piloted, field researchers described the other systems (with pros and cons) with pictures to girls, teachers and janitors during NGDs. Therefore, the ranking of the disposal systems was based on description and pictures provided by the field researchers, only. This was only a limitation for the chute disposal system, as most participants were familiar with the three temporary disposal systems. Nevertheless, participants were still able to comment on the appearance and anticipated benefits and challenges of all the options. We conducted NGDs with teachers and janitors as there were not enough people from each category in each school to organize true focus groups. Despite the heterogeneous nature of the participants, we observed good levels of participation from all. We implemented the chute disposal systems in implementation schools for 6 months (presented separately) where more schoolgirls disposed of the disposable pad during the last menstrual period in the chute disposal system (55% vs 62%, APD: 8 (− 1, 18). During this time, the disposal pit did not fill-up. As a result, we do not have data about exact filling time and ultimate disposal of the disposed products. However, the chute disposal system is estimated to fill up within 2–3 years (DSK, personal communication), so it requires periodic follow up to check when it needs to be emptied. During our six-month period of implementation, we were still able to identify potential maintenance issues that may contribute to emptying needs, over time.

Finally, there was an annual period of examinations and vacation in schools in the middle of our implementation period, during which girls spent fewer hours in school than usual. As a result, we may have been limited in collecting data on typical usage of chute disposal systems, as many girls did not use the disposal system during exam and vacation.

## Conclusions

Girls in LMICs like Bangladesh lack adequate facilities in schools to facilitate changing and disposing of menstrual absorbents. The lack of disposal systems impedes girls’ comfort to attend school and impairs toilet functionality. Consequently, optimal menstrual hygiene management in schools becomes difficult and can affect school absence or the inability to change menstrual products during school hours. Providing a disposal system that does not require frequent emptying can be a sustainable option to facilitate changing and disposal of menstrual waste in schools, facilitating girls to manage their menstruation effectively. The chute disposal system is a promising option to meet this need, although regular supervision and an adequate management plan for operating the system long term needs to be explored in this setting. Improvement in enrollment and attrition of girls in schools is a priority issue [40–43], therefore, menstrual hygiene management facilities in schools should be considered ahigh priority issue, as well.

## Supplementary information

**Additional file 1.** Instruments used for data collection in the study. This additional file contains different data collection instruments used in the different part of the study. The guidelines have been listed below: Instrument 1A: In-depth interview guideline for girl students (Pre-intervention phase). Instrument 1B: In-depth interview guideline for boy students (Pre-intervention phase). Instrument 1C: Key informant interview guideline for janitors in the schools (Pre-intervention phase). Instrument 1D: Participatory activities, drawing exercise and vignette approaches to explore MHM intervention (Pre-intervention phase). Instrument-2A: Semi-structured interview with user girls for disposal system (Intervention development and initial piloting phase). Instrument-2B: Interview with responsible persons for disposal system (Intervention development and initial piloting phase). Instrument 3A: Fidelity Assessment of chute disposal system Implementation among Urban and Rural Schools (Intervention implementation phase). Instrument 3B: Interview of girls to explore experience, perceptions and current practices regarding chute disposal system (intervention implementation phase).

## Data Availability

Institutional Review Board approval was obtained for public sharing and presentation of data on a group level only. To maintain participants’ anonymity and confidentiality, the data set generated during the study will not be shared.

## Abbreviations

MHM: Menstrual Hygiene Management
LMIC: Low- and Middle-Income Countries
WASH: Water, Sanitation and Hygiene
icddr,b: International Center for Diarrhoeal Disease Research, Bangladesh
DSK: Dushtha Shasthya Kendra
IDI: In depth interview
KII: Key informant interview
NGD: Natural group discussion
