# Supplementary material for: Piloting an acceptable and feasible menstrual hygiene products disposal system in urban and rural schools in Bangladesh
Source: BMC Public Health. 2020 Sep 7;20:1366. doi: 10.1186/s12889-020-09413-x (PMC7487504; doi:10.1186/s12889-020-09413-x)
Supplement: Supplementary file 1 — Additional file 1. Instruments used for data collection in the study. This additional file contains different data collection instruments used in the different part of the study. The guidelines have been listed below: Instrument 1A: In-depth interview guideline for girl students (Pre-intervention phase). Instrument 1B: In-depth interview guideline for boy students (Pre-intervention phase). Instrument 1C: Key informant interview guideline for janitors in the schools (Pre-intervention phase). Instrument 1D: Participatory activities, drawing exercise and vignette approaches to explore MHM intervention (Pre-intervention phase). Instrument-2A: Semi-structured interview with user girls for disposal system (Intervention development and initial piloting phase). Instrument-2B: Interview with responsible persons for disposal system (Intervention development and initial piloting phase). Instrument 3A: Fidelity Assessment of chute disposal system Implementation among Urban and Rural Schools (Intervention implementation phase). Instrument 3B: Interview of girls to explore experience, perceptions and current practices regarding chute disposal system (intervention implementation phase). [file 12889_2020_9413_MOESM1_ESM.docx]

# Instrument 1A: In-depth interview guideline for girl students (Pre-intervention phase)

**Introductory Session**

Introduce researchers themselves & objective of study & interviews as clearly as possible to participants followed by ethical issues. Ask if they are comfortable to share us puberty/menstruation/menstrual hygiene management related issues. Allow them enough time & environment to feel comfortable to work with us. Play games with them for Ice breaking & ensure an ambient environment. Let the interviewee know that this conversation will be recorded only if he/she permits, and collect the socio-demographic information.

## Part 1: Perception and practices about menstruation, puberty and embarrassment

##

**Why we need this information**

- Exploring existing perceptions and practices around menstruation will help developing behaviour change materials and providing menstrual hygiene management facilities to supportive school environment for girls to retain them in school by.

**Questions**

1. What do you know about puberty, menstruation and menarche? Please describe how did you come to know these (*probe: text books, parents, relatives, friends, mass media, elderly people, neighbours, peer groups*)?
2. Did you reach your puberty and menarche? If yes, when and how did you notice that (age of first menstruation, place of first menstruation)? How did you manage your menarche? What related events happened to you during that time (*Probe: shocks, surprise, shame, guilty, good or bad feelings, stigma etc.*)?
3. Did you know about puberty and menstruation before you reached menarche? If no, why (*Probe: family environment, stigma, ignorance, taboo)?* If you would have known earlier then what would be the benefit? If yes, what, how and from whom (*probe: parents, relatives, friends, mass media, text books, elderly people, neighbours, peer groups)?*
4. What do you know what happens when a boy reaches puberty (*Probe: shocks, surprise, shame, guilty, good or bad feelings, stigmatized etc*)? [Ask from her brother or any other male family members, source of information regarding this]? What can be done to minimize/ manage these for those who didn’t reach puberty and menarche yet?
5. How do you manage your menstruation currently (in or outside home including school, travelling period, staying outside/relatives’ house)? What do you use during menstruation (*probe: differences in different time and place*)? How and why? Please describe in details (*repeated use, cleaning, handwashing, drying, storage, disposal, carry emergency products/deal with unexpected situations*).
6. Do you need to spend any amount of money to manage your menstruation? If yes, how much? How do you manage expenditure for menstrual hygiene management? (Probe*: who buy the products, who pay, any problem faced anything else)*.
7. What are the perceptions around menstruation/puberty among surrounded people (*Probe: stigma, teasing in school, home and community*).
8. Do girls talk about menstruation/ puberty at school? Do boys and teachers talk about such issues? If yes, what are those? Can you please share us any experience regarding this?
9. Have you ever experienced or seen any girl is being teased or stigmatized about anything including menstruation by any boy students, other girls or teachers in the school compound? Please explain?
10. Do you come to school during menstruation? Do you face any barrier to attend school during menstruation (*physical, social, religious, institutional*)? Why? Please describe with example. If so, what can be done to reduce such barriers?
11. Sometimes girls may not come to school when they are menstruating. Have you heard of girls who do not come to school while they are menstruating? What about you? What reasons do you think you/girls have for not coming to school while they are menstruating?
12. Do you think it is necessary for boys to have knowledge about girls’ puberty/menstruation and vice versa? If yes, why and how (*Probe: teaching them during classes, boys and girls combined session or separate, including any topic in study curriculums, identifying teachers and teaching process etc*)? If no, why not?

##

## Part 2: Facilities for menstrual hygiene management at school compound

##

**Why we need this information**

- We want to build upon existing structure in the school for which we need to know the current barriers and facilities that needs to be modified, changed or improved to best fit with the proposed interventions.

**Questions**

1. How do you feel about your school toilet? Are they currently functional? If not, why (Probe: if any toilet is locked, how do they manage menstruation)? Does it contain any slang language? How do you feel when you find such thing inside the toilet (who write)? How this issue can be solved?
2. Is there any separate room/private place in your school to menstrual hygiene management, change disposable pad/cloth during menstruation? Where? Do you have access? If not, what do you do during menstruation?

1. Is there sanitary pad disposal bins/place in the girl's latrine/ changing room/school compound? If yes, what? If not what do you do?
2. Do the school have enough water, soap for handwashing and menstrual hygiene product supply for menstrual hygiene management? If yes, describe? If not, how do you manage menstrual hygiene in the school? How it can be improved? (Probe: if they bring products in school bag, if janitors sell etc).
3. Is there any other problem you face during menstruation in the school compound?

##

## Part 3: Source of information, social support, infrastructure/products

**Why we need this information**

- We want develop behaviour change communication strategy to improve menstrual hygiene knowledge and practices in the school compound for which we need to understand about current curriculum content, information provided and support system available to best fit/explore the proposed intervention.

**Questions**

1. What information, advice have you previously received on these topics? How? Through whom? (*Probe: parents, family members, curriculum, extra-curricular, media, friends, teachers etc*).
2. Please explain some of the information or advices that you still remember or practice? What does your school text books explains about menstruation and puberty? In which class specifically? Did you learn about these issues before menarche? Who teaches these classes? (*Probe: male or female teachers*). How do you feel learning such issues from male teachers?
3. How would you prefer to receive information on these topics that can help to continue your habit to menstrual hygiene management (who will teach, how and with what information)? What and how much materials/information currently exists on these topics in school curriculum? If any information exists, to what extent is this sufficient? Why or why not? What role do you think your friends, family members and teachers can play in your regular menstrual hygiene management practices?
4. What products if any (e.g. disposal bin, privacy, pads, space, soap, water, basin, ) presently exist at school compound to support menstrual hygiene management? (*Probe if they mention the presence of any related products: to what extent is this sufficient? Where are these products located if any exists? What kinds of improvements or modifications are required?*)
5. What kind of motivators do you think would help you or your fellow students to practice proper menstrual hygiene management? (*Probe: kinds of messages, monitoring, reminders, rules, cue cards, media etc*)?
6. Sometimes people know about behaviour and have been trained in it but they still continue to practice their old behaviour *(Probe: the use of unhygienic rags/cloths etc*). Can you tell me why this might happen? How can we help them to practice the recommended behaviour?
7. What is your opinion if we form gender clubs *(including girls and boys to discuss gender equality and sensitivity issues separately under teacher’s supervision*) to discuss puberty and menstrual hygiene management issues? Please describe.

# Instrument 1B: In-depth interview guideline for boy students (Pre-intervention phase)

**Introductory Session:**

Introduce researchers themselves & objective of study & interviews as clearly as possible to participants followed by ethical issues. Ask if they are comfortable to share us puberty/ menstruation/menstrual hygiene management related issues. Allow them enough time & environment to feel comfortable to work with us. Let the interviewee know that this conversation will be recorded only if he/she permits, and collect the socio-demographic information.

## Part 1: Perception and practices about puberty and menstruation

**Why we need this information**

- We aim to promote supportive school environment for girls and gender sensitivity in the school compound for which exploring male students' perceptions is important.

**Questions:**

1. What do you know about puberty (boys and girls)? Please describe? How did you come to know these (Probe: text books, parents, relatives, friends, mass media, elderly people, neighbours, peer groups)?
2. Do you think that you have reached puberty? If yes, when and how did you notice that? What related events happened to you during that time (Probe: body and mood changes special feelings, *shocks*, shame, stigma etc.)?
3. Did you know about puberty before you reached your own? If no, why (Probe: family environment, stigma, ignorance, taboo)? If you would have known earlier then what would be the benefit? If yes, what, how and from whom (probe: parents, relatives, friends, mass media, elderly people, neighbours, peer groups)?
4. What do you know about what happens with girls when she reaches puberty (Probe: menstruation, shocks, surprise, shame, guilty, good or bad feelings, and stigma)? [Ask his experience of his sister or any other female family members]? How do you feel?
5. How do the girls in your school or your female family members at home manage their menstruation? Do you know what do they use during menstruation? Please explain? What are the perceptions around puberty/ menstruation among your fellow friends, school girls, school boys, teachers and parents, relatives, community people? (Probe: stigma, teasing etc).
6. Do boys talk about menstruation/ puberty at school? If yes, what are those? Can you please share us any experience regarding this?
7. Do girls and teachers talk about menstruation/ puberty at school? If yes, what are those? Can you please share us any experience regarding this (class lectures, seeking leave during menstruation, sudden staining incidence)?
8. Do you have any experience of girls being teased or harassed due to menstruation in the school compound (by girls, boys, teachers, janitor)? Please explain?
9. What do you think/know, do the girls come to the school during menstruation? Do they face any barrier to attend school during menstruation (physical, social, religious, institutional/ lack of school facilities)? If yes, why? Please describe with example. If so, what can be done to reduce such barriers?
10. Sometimes girls may not come to school when they are menstruating. Have you ever heard of any girl student of you school or your sister/female friend missed school due to menstruation or any sickness? Please describe?
11. Do you think it is necessary for boys to have knowledge about girls’ puberty/menstruation and vice versa? If yes, why and how (Probe: teaching them during classes, boys and girls combined session or separate, including any topic in study curriculums, identifying teachers and teaching process etc)? If no, why not?

## Part 2: Facilities for menstrual hygiene management at school compound

**Why we need this information**

- Existing facilities are important to support effective MHM interventions. Some schools provide same toilet facilities for girls. If we aim to provide hardware for MHM, we need to explore boys' voices and expectations.

**Questions**

1. How do you feel about your school toilet (probe: user friendly or not, cleanliness, access to toilet whenever need, water supply, bin, handwashing facilities? Is it functional and open for all? If not, why (Probe: if any toilet is locked, how do the girls in school manage their menstruation)? How it can be improved?
2. Have you ever seen anything written on the walls of the school toilets? If yes, what are those? Were there any unpleasant words/slangs written to tease girls? Why? What do you think about that or how would girls feel about that when using the toilets? How it can be improved?
3. Do the toilets contain menstrual hygiene management facilities/ disposal bins? If yes, how do you feel about that? Please describe.
4. Is there any separate room/private place for girls in your school to menstrual hygiene management? Where? Do girls have access? If not, where and how do they manage menstruation at school? How do you feel about that?
5. Is there any other barrier, you think, girls face during menstruation in the school compound?
6. Did you find anything such as used sanitary napkins, cloths inside the toilet? If yes, how do you feel when you find such thing inside the toilet? Please describe?
7. What do you think, boys and girl’s toilet should be combined or separated? Why or why not (try to understand if there is any social, individual, gender related barriers)?

## Part 3: Source of information, social support, infrastructure/products

**Why we need this information**

- We want to develop behaviour change communication strategy to improve menstrual hygiene knowledge and practices in the school compound for which we need to understand about current curriculum content, information provided and support system available to best explore a gender sensitive intervention.

**Questions:**

1. What does your school text books explains about puberty and menstruation? In which class specifically? Who teaches these classes and how (Probe: boys and girls combined class, male or female teachers)? From whom (male or female teacher) and how (combined class, separate session, cultural class) would you like to learn such issues?
2. Do you think the existing curriculum content on these topics is enough to form proper knowledge and good practices among students? Why or why not?
3. Do you think the existing curriculum content on these topics is enough to reduce gender barriers and stigma among boys and girls and also among teachers? Why or why not?
4. Who can play role in your school to promote menstrual hygiene management knowledge and practices (your friends, family members and teachers)? How (Probe: kinds of messages, monitoring, reminders, rules, cue cards, media etc?
5. Do you know if there are any products (e.g. disposal bin, privacy, pads, space, soap, water, basin, water storage vessel, deep tube wells etc) presently exist at school compound to support menstrual hygiene management of the girls? (Probe if they mention the presence of any related products: to what extent is this sufficient? Where are these products located if any exists? What kinds of improvements or modifications are required?)
6. If your sister attended this school, what facilities would you think be adequate for her to attend school during her menstruation?

# Instrument 1C: Key informant interview guideline for janitors in the schools (Pre-intervention phase)

## Section-1: Daily school activities

1. Please tell me about yourself (name, job duration in the school, recruitment, salary) and about what do you do here in the school (daily activities)?
2. How much you earn as a janitor of this school? Do you feel it is okay to run your family and other expenditures? If not, why? What can be done?
3. How much you have to do cleaning activities (Sweeping, washing, disposing) and what areas (classrooms, toilet, water sources, bins)
4. How frequently you clean these (probe for each of the areas)? With what materials and agents, you use to clean these?

## Section-2: Menstrual hygiene management practices in the school

1. What are the practices of the girls and female teacher as well as of herself regarding menstrual hygiene management in school? How do they and you manage menstruation here?
2. How do you feel about the current menstrual hygiene management facilities in this school? Do you think that the facilities available in this school are enough for them to stay in the school during their menstruation? Why or why not?
3. Do you have to clean and dispose products/pads used by the girls and female teachers during their menstruation in the school? If yes how do you do that (frequency of disposing, place of disposing, any disgust feeling etc.)? If no, who does it and how?
4. Have you ever seen boys to tease girls regarding menstruation and any other issues? Please explain form your experiences? What can be done to reduce that?

## Section-3: Recommendation for the intervention

1. Is/was there any intervention related to water supply, handwashing or menstrual hygiene management in this school? Please describe (hardware such as bins pads; software such as sessions, guidebooks, pictures etc.)? Was there any role for you? Did you require maintaining anything? How? How did you feel (like and dislike)? Why?
2. What can be done to improve menstrual hygiene management facilities in the school and inside the latrine? How (probe for hardware including disposal procedures and place, stock up of sanitary products, cleaning agents and software)?
3. If we initiate an intervention in your school what role you can play (how can we involve him/her in the maintenance committees, willingness to dispose used menstrual products from bins, way to stock up menstrual products)?
4. What do you think how the boys would react to our intervention programs for girls menstrual hygiene management?

# Instrument 1D: Participatory activities, drawing exercise and vignette approaches to explore MHM intervention (Pre-intervention phase)

## Part 1: Participatory activities and drawing exercise

##

**Why we need this information**

- We aim to develop a novel intervention to support girls to manage menstruation at school compound for which girls' voices needs to be heard. Games, quizzes and participatory exercises better explores their views.

**Questions**

1. Please draw a school environment that you think supportive for you to attend school during menstruation (individual will draw using paper and color pencil will be supplied with enough space to draw).
2. Now we will show you some pictures of menstrual hygiene management amenities including disposal bin, pads/rags etc. After that we will discuss with you regarding this issue and will ask you to rank which one you prefer. *(This activity will be recorded in the form of notes).*

## Part-2: Vignette approaches

1. **Story 1:** Rina reads in grade 8 in a co-ed school. She had a class on puberty and menstruation on last Monday; however, her class teacher Rahima Khatun did not come. They had exam close, thus their head teacher Mr. Zaker Ali came to take the class. When he started discussing the topic, he found that some students were leaving the class making excuse for going to wash room. What do you think what happened to them? Why? What can be done?
2. **Story 2:** Sabiha reads in grade 6 and she is a class topper. One day she found that she is bleeding and stained the cloth and school bench. She was scared and did not know what to do? Besides, all her class fellows were teasing her. She could not share her problem with anyone as she was afraid and she did not know whom to ask help? She left school and did not come again. What do you think what happened to her? Why? What could be done to help her? Why she did not come back to school again? How her study was affected?
3. **Story 3:** One day while you were playing, you found that your class captain Sabbir and Rosy was talking about puberty and menstruation. Sabbir shared I have been watching the new TV advertisement that says to speak about menstruation and it is not a shame. I think this was good to educate all. How would you feel about the topic they were discussing? How would you feel about discussing the topic among a boy and a girl?
4. One day Roni went to the school toilet which is shared by both girls and boys. He found the disposal bin was filled with used sanitary pads with blood. After coming out from the toilet he started discussing about the issue with his friends. What do you think what Roni and his friends were discussing about the issue? Why they were discussing in this way? What can be done and how?

# Instrument-2A: Semi-structured interview with user girls for disposal system (Intervention development and initial piloting phase)

| 1. Id: \|__\|__\|__\|__\| b. Date of visit: ______________ c. Name of visit: _____________ 2. Type of disposal system: 1. Paddle bin. 2. Swing top bin 3. Bucket with lid 4. Chute disposal system 3. Type of respondent: 1. Girl student 2. Boy student 3. Teacher 4. Janitor 4. a. Respondent’s name: ____________________________ b. Contact Number:   c. Grade: d. Section: e. Age: d. Sex:  f. Roll: g. Address:   1. Facilitator: 1. Name:______________________ 2. Designation:________________3. Sign:__ |
| --- |

## Introductory statement

- We are from icddr,b. We came to you to hear about the experiences you have had with the MHM Product since we provided here. We want to learn what did you like or dislike about it.

1. Is there any disposal bin in your school? If yes, where the disposal bins are installed/located?

|  |
| --- |

1. Do you use it? If yes, why? Usually what type of waste you dispose here and how often?

|  |
| --- |

1. Have you seen anyone in your school (friends/classmate) using it? What purposes, please detail?

|  |
| --- |

1. Do you know who maintain disposal bin? How (Frequency and place of final disposal and process)?

|  |
| --- |

1. Do you like the disposal system? Why or why not (color, size, cover, process, difficulty, ease, disgust)?

|  |
| --- |

1. Do you think the current location of the disposal system is ok? Why or why not? What is your suggestion for placing the system?

|  |
| --- |

1. Do you have any idea on modification disposal system? Please detail?

|  |
| --- |

1. What is your opinion about the activities we conducting in your school? Please detail?

|  |
| --- |

1. Do you have any suggestion to improve the way of our activities? Please detail?

|  |
| --- |

# Instrument-2B: Interview with responsible persons for disposal system (Intervention development and initial piloting phase)

| 1. a. School code: \|__\|__\|__\|__\| b. Date of visit: _____________________ 2. Type of disposal system: 1. Paddle bin. 2. Swing top bin 3. Bucket with lid 4. Chute disposal system 3. Type of respondent: 1. Girl student 2. Boy student 3. Teacher 4. Janitor 4. a. Respondent’s name: ____________________________ b. Contact Number:   c. Grade: d. Section: e. Age: d. Sex:  f. Designation: g. Address:   1. Facilitator: 1. Name:______________________ 2. Designation:________________3. Sign:__ |
| --- |

## Introductory statement

- We are from icddr,b. We came to you to hear about the experiences you have had with the MHM Product since we provided here. We want to learn what did you like or dislike about it.

1. What is your view about the disposal system we have provided in your school? Please describe?

|  |
| --- |

1. How frequently the disposal bin is cleaned? Who, when, where and how is disposed and maintained?

|  |
| --- |

1. Was there any difficulty during disposing used product/waste from bins finally?

|  |
| --- |

1. Did you like the disposal system provided by icddr,b? Why or why not?

|  |
| --- |

1. Do you anything to say on further development/ modification of the disposal system (price, availability, material, use, and feasibility) and school-based management?

|  |
| --- |

1. What is your view regarding the activities that we are conducting in this school?

|  |
| --- |

1. Do you have anything to say about how to best motivate girls to practice MHM behaviors in school (BCC materials such as, poster, instruction sheet, cue card, CHP’s visits or anything else?)

|  |
| --- |

# Instrument 3A: Fidelity Assessment of chute disposal system Implementation among Urban and Rural Schools (Intervention implementation phase)

## Section 1 (Questionnaire identification)

1. Questionnaire identification number

School Code................................................

## Section 2 Spot Check

2.1 Condition of the disposal system?

Condition of the disposal system(Multiple responses are allowed here)

Lids working properly (Yes = 1, No = 0)

System working properly (Yes = 1, No = 0)

Foul smell (Yes = 1, No = 0)

Area surrounding the lid maintained clean (Yes = 1, No = 0)

Blood stains on the chutes(Yes = 1, No = 0)

Jammed pad inside the chute (Yes = 1, No = 0)

Other: specify.......77 (Please specify ……………………………………….)

Condition of the cue card?

1. Provided number………, Present number……………..
2. Intact …………………..(In number), Damaged ……………….(In number)

Q 2.2.3General Toilet Facilities (Multiple responses are allowed here)

1. Water supply in toilets(Yes = 1, No = 0)
2. Water supply for handwashing (Yes = 1, No = 0)
3. Hand washing soap (Yes = 1, No = 0)
4. Cut pieces for paper (Yes = 1, No = 0)
5. Bad smell (Yes = 1, No = 0)
6. Adequate light (Yes = 1, No = 0)
7. Properly working door lock (Yes = 1, No = 0)
8. Other: specify.......77 (Please specify ……………………………………….)

## Section 3: Interview of the students for assessment of usage of Disposal System

Q.1 Name of respondent: ............................................................

Q .2 Age of respondent: Year Month

Q.3 Educational status of mother of the respondent (If respondent say don’t know then use code “99”)] …………………..

Q .4 Educational status of father of the respondent (If respondent say don’t know then use code “99”)].....................

Q.5 Main occupation of father of the respondent

Q.6 Main occupation of mother of the respondent

| **[Occupation Code]:**  01. Homemaker for own HH/Housewife]  02. Labor (physical labor)  03. Salaried job  04. Mason/Carpenter/Electrician/Plumber  05. Van/Rickshaw puller/battery driven auto rickshaw driver  06. Cobbler/maker  07. Shopkeeper/Business/ambulant vendor]  08.Tailor (both in home and shop)] | 9. Driver  10. Cottage industry  11. Garment worker  12. Domestic maid /servant  13. Beggar  14. Unemployed/ Disabled  15. Died/untraced  16. Staying abroad  17. Doctor  18. Farmer  77. Others (specify)]______________  99. Don’t know |
| --- | --- |

Q.7 Did you dispose of your sanitary pads at school? (Yes = 1, No = 0),

If answer is NO, please specify the reason and your disposal location and end the interview

Q.8 If yes, did you dispose it at icddr,b disposal system? (Yes = 1, No = 0)

If No, please explain the reason....................................................................................................................................................

Q.9 If answer of Q .8 is Yes, how did you dispose the pads?

............................................................................................................................................

Q.10 Do you have any problems using this disposal system?(Yes = 1, No = 0), if answer is NO end the interview

Q .11.1 what kind of problems do you face? (Multiple responses are allowed here)

1. Long queue in the toilet (Yes = 1, No = 0 )
2. Broken lid (Yes = 1, No = 0 )
3. Toilets are locked (Yes = 1, No = 0 )
4. Toilets are unusable (Yes = 1, No = 0 )
5. Stinking disposal inlet (Yes = 1, No = 0 )
6. Blocked disposal chute (Yes = 1, No = 0 )
7. Blood stain on the lid (Yes = 1, No = 0 )
8. Others ….77 please specify …………………………………………

Q.11.2 How did you overcome the problem? ……………………………………………………………………

……………………………………………………………………………………………………………………………………………

Q.15How can we improve these barriers?

……………………………………………………………………………………………………………………………………………..

# Instrument 3B: Interview of girls to explore experience, perceptions and current practices regarding chute disposal system (intervention implementation phase)

101. Could you please share your experience of using the disposal system with us? (Probe: Both positive and negative experiences, process of using: wrapping before disposing, availability of newspaper pieces, management if newspaper cutting were unavailable)

102. What are the advantages of having chute disposal system in school? What do you think? Please give us an example. (Probe: Easy to use, effective, time saving, durable, hygienic, clean, well maintained, brought change in the school environment, comfort in attending classes during menstruation)

103. Have you ever faced any challenges/barriers in using the disposal system? If YES, could you please share your experience with us? (Probe: Position of chute, location, unhygienic toilet, bad odor, improper disposal by other girls/blockage of system, unclean lid, lack of light in toilet, fear of dark inside the chute, feeling of disgust, discomfort, solving the problems)

104. Will you suggest for any modification of the existing disposal system (e.g., lights to avoid darkness)?
